# A Label-Free Electrochemical Impedimetric Immunosensor with Biotinylated-Antibody for SARS-CoV-2 Nucleoprotein Detection in Saliva

**DOI:** 10.3390/bios12050265

**Published:** 2022-04-22

**Authors:** Ching-Chou Wu, Yu-Huan Chiang, Hsin-Yu Chiang

**Affiliations:** 1 Department of Bio-industrial Mechatronics Engineering, National Chung Hsing University, Taichung 402, Taiwan; fs3291940@hotmail.com; 2 Innovation and Development Center of Sustainable Agriculture, National Chung Hsing University, Taichung 402, Taiwan; 3 ACK Biotechnology Co., Ltd., Taichung 402, Taiwan; exlenv6@acuchecks.com

**Keywords:** SARS-CoV-2, nucleocapsid protein, electrochemical impedance spectroscopy, immunosensor, saliva, antigen tests

## Abstract

The timely detecting of SARS-CoV-2 coronavirus antigens for infection validation is an urgent request for COVID-19 pandemic control. This study constructed label-free electrochemical impedance spectroscopy (EIS)-based immunosensors based on gold nanostructured screen-printed carbon electrodes (AuNS/SPCEs) to detect the SARS-CoV-2 nucleocapsid protein (N-protein) in saliva. Using short-chain 3-mercaptopropionic acid (MPA) as a linker to covalently bond streptavidin (SA) and bovine serum albumin (BSA) for controlling the oriented immobilization of the biotinylated anti-N-protein antibody (BioAb) can offer a greater sensitivity, a lower limit of detection (LOD), and better reproducibility of immunosensors (defined as BioAb/SA-BSA/MPA/AuNS/SPCEs) than the antibody randomly immobilized immunosensors and the long-chain 11-mercaptoundecanoic acid (MUA)-modified immunosensors (BioAb/SA-BSA/MUA/AuNS/SPCEs). The BioAb/SA-BSA/MPA/AuNS/SPCE-based immunosensors presented good linearity from 0.01 ng/mL to 100 ng/mL and a low LOD of 6 pg/mL in a phosphate buffer solution (PBS) and PBS-diluted saliva. Moreover, the immunosensor exhibited little cross-activity with other viral antigens such as MERS-CoV N-protein, influenza A N-protein, influenza B N-protein, and SARS-CoV-2 spike protein, indicating the high specificity of the immunosensors. The disposable label-free EIS-based immunosensors have promising potential in facilitating the rapid and sensitive tests of saliva-based COVID-19 diagnostics.

## 1. Introduction

Since the coronavirus disease 2019 (COVID-19) was declared by the World Health Organization (WHO) in 2020, the cumulative death toll is over six million to date [1]. The COVID-19 pandemic, caused by the severe acute respiratory syndrome coronavirus 2 (SARS-CoV-2), has induced a catastrophic impact on the human healthcare system and economy [2]. The symptoms of COVID-19, from mild to severe, including fever, cough, headache, loss of taste or smell, sore throat, and pulmonary infiltrates, may appear 2–14 days after exposure to the virus [3]. However, some infected patients are asymptomatic. These asymptomatic patients still have a great potential to spread the COVID-19 disease to other people. Although vaccination can reduce the risk of infection and severe symptoms of COVID-19 against viral transmission, the breakthrough infection and the asymptomatic infection still occur by an emerging variant virus [4]. Therefore, developing a rapid, sensitive, and accurate detection platform to diagnose COVID-19 carriers is crucially essential for preventing the disease from spreading at the early stages.

COVID-19 diagnostic testing can be classified into two categories [5,6,7,8,9]: the viral tests, including antigen tests and nucleic acid amplification tests (NAATs), and the antibody (Ab) tests, containing IgG and IgM antibodies of anti-SARS-CoV-2 proteins. Generally, serum Ab detection determines past infection and vaccine effectiveness [5,6]. Viral tests are adopted to confirm the current infection cases [5,6]. NAAT techniques, such as reverse transcription-polymerase chain reaction (rtPCR) [10,11] and reverse transcription loop-mediated isothermal amplification [12], are the gold standard to confirm SARS-CoV-2 carriers and variant viruses. After collecting samples through a nasopharyngeal swab [11,12], the laboratory-based NAATs need trained persons to perform virus lysis and nucleic acid extraction, limiting the NAATs in developing a point-of-care (POC) device. Antigen detection based on an affinity reaction, such as the spike (S) protein (S-protein) [13,14,15,16,17,18], the S-protein receptor-binding domain (RBD) [19,20], and nucleocapsid protein (N-protein) [21,22,23,24,25,26], is popularly used for the diagnostics of early-stage COVID-19 disease. Compared to the NAAT-based assay, the operational procedures and testing time of viral antigen diagnostics are easy and rapid (within 60 min) [21,22,23,24,25,26].

Several rapid antigen diagnostic techniques are conducted for POC testing. The lateral flow immunochromatographic assay (LFIA) is the most popular in vitro diagnostics product approved by Emergency Use Authorization (EUA) by the U.S. Food and Drug Administration for COVID-19 detection [27,28]. LFIA strips for SARS-CoV-2 N-protein have excellent specificity (approximate 100%) but ordinary sensitivity (about 80%) [29], which is suitable for the rapid qualified detection of COVID-19 disease [30]. Biosensors integrating specific recognition molecules with sensitive transducers have attracted wide attention for developing sensitive POC devices [13,14,15,16,17,18,19,20,21,22,23,24,25,26]. Among different transducers, electrochemical detectors possess a promising potential to construct POC biosensing platforms due to good compatibility with portable electrical readers, ease of large-scale electrode production, and well-developed immobilization techniques of recognition molecules on various electrodes. Different electrochemical measuring strategies, including amperometry [23,31], differential pulse voltammetry (DPV) [17,24,32], square wave voltammetry (SWV) [25,33], and electrochemical impedance spectroscopy (EIS) [34,35,36,37], have been adopted in affinity biosensors to quantify the concentration of SARS-CoV-2 antigens, antibodies, and nucleic acid fragments. EIS is a sensitive label-free sensing technique that can directly quantify the change of an affinity reaction in the electrode/electrolyte interface [38]. Lorenzen et al. immobilized the recombinant N-protein of SARS-CoV-2 on the poly(3,4-ethylenedioxythiophene)/Au nanoparticle (AuNP)-electrodeposited steel mesh electrodes to detect the anti-N-protein Ab of serum samples [34]. Avelino et al. deposited polypyrrole and AuNPs on a tin-dopped indium oxide electrode as a nanostructured conductive substrate to construct a genosensor to detect the N-protein gene [35]. Muñoz and Pumera developed a 3D-printed graphene-based electrode with the RBD immobilization for detecting the S-protein-spiked serological samples in an indirect competitive immunoassay [36]. Rashed et al. integrated RBD recombinant protein onto the commercial Au electrode array for detecting SARS-CoV-2 antibodies [37]. Among the SARS-CoV-2 antigens, the N-protein is the most abundant protein with ~1000 copy numbers per viral particle and is highly conserved, unlike easy mutation of the S-protein [39]. The N-protein can be detected up to 1 day before clinical symptoms appear [25]. Thus, the N-protein has promising potential to be a specific biomarker for COVID-19 diagnosis. To the best of our knowledge, few studies have explored the sensing properties of EIS-based N-protein immunosensors in saliva samples. Nasopharyngeal or oropharyngeal swap sampling is a semi-invasive specimen collection that causes discomfort in testers. In contrast, salivary detection permits noninvasive sampling, which has great potential as an alternative method for rapid COVID-19 screenings [10,11,17,22,31,39].

Generally, Au nanostructures (AuNS) or AuNP-deposited electrodes can effectively increase the sensitivity of EIS-based sensors due to the increased surface roughness and conductivity [38,40,41]. Therefore, this study proposed the construction of an EIS-based N-protein immunosensor based on the AuNS-deposited screen-printed carbon electrodes (SPCEs). The AuNS/SPCEs were first immobilized with a mixture of streptavidin (SA) and bovine serum albumin (BSA) and then interacted with biotinylated anti-SARS-CoV-2 N-protein Ab (BioAb) to form the SARS-CoV-2 N-protein immunosensor. The Ab immobilization strategies were compared to optimize the sensing properties. Moreover, the detecting ability of immunosensors in the N-protein-spiked saliva samples was explored in detail.

## 2. Materials and Methods

### 2.1. Reagents and Chemicals

Mouse monoclonal anti-SARS-CoV-2 N-protein Ab (Cat. RM3127-00) and N-protein (Cat. CG101-00) were obtained from Vazyme. SA was purchased from BioVision (MW:53 kDa, Cat. 7936). A biotinylation chemical, EZ-Link Sulfo-NHS-LC-Biotin, was purchased from Thermo (Cat. 21335). SARS-CoV-2 S-protein (Cat. 61831) was obtained from Leadgene Biomedical. MERS-CoV N-protein (Cat. GTX135663-pro), influenza A virus N-protein (Cat. GTX135868-pro), and influenza B virus N-protein (Cat. GTX135867-pro) were bought from GeneTex. Potassium hexacyanoferrate(II) trihydrate (K_4_[Fe(CN)_6_]) 3H_2_O and potassium hexacyanoferrate(III) (K_3_[Fe(CN)_6_]) were purchased from Showa. 3-Mercaptopropionic acid (MPA), 11-mercaptoundecanoic acid (MUA), N-(3-dimethylaminopropyl)-N′-ethylcarbodiimide hydrochloride (EDC), N-hydroxysuccinimide (NHS), 2-(N-morpholino) ethanesulfonic acid (MES), gold(III) chloride trihydrate (HAuCl_4_), potassium chloride (KCl), polyethylene glycol sorbitan monolaurate (Tween 20), artificial saliva for pharmaceutical research (Cat. SAE0149), glycine, and BSA (MW: 66 kDa) were purchased from Sigma-Aldrich. The 10 mM MPA solution was prepared in double-distilled water. Phosphate buffer solution (PBS, pH 7.4) was prepared by 10 mM NaH_2_PO_4_ and 10 mM Na_2_HPO_4_ and used in all immune experiments. The three-electrode-type SPCEs, consisting of a carbon working electrode (WE) of 0.071 cm^2^ area, a carbon counter electrode, and a silver pseudo-reference electrode (RE), were obtained from Zensor R&D (TE100). All chemicals were of reagent grade and were used without further purification. All solutions were prepared with water purified through a Milli-Q system.

### 2.2. Immunosensor Preparation

The AuNS electrodeposition procedure of SPCEs refers to our previous studies [40,41,42]. Initially, the SPCEs were cleaned by a cyclic potential from 0 to 1.3 V for 20 cycles with a 0.1 V/s scanning rate and then oxidized at 2.0 V for 30 s in 0.1 M PBS (pH 7.0) with a three-electrode system using a Pt plate as the counter electrode and a commercial Ag/AgCl electrode as the RE. These procedures can increase the hydrophilicity and electron transfer rate of SPCE surfaces. Subsequently, the oxidized SPCEs were dipped in the 100 mM KCl-containing HAuCl_4_ solution (8 mM, pH 2.0) for the two-step AuNS deposition. The first step was AuNP nucleation on the oxidized SPCEs using a cyclic potential from 0.5 to −0.5 V with a scan rate of 50 mV/s for seven cycles. The second step was AuNS formation on the AuNPs with a step potential at 0.62 V for 10 min. The AuNS/SPCEs were rinsed with double-distilled water to remove the free ions from the electrode surface for subsequent surface modification.

**Scheme 1:**
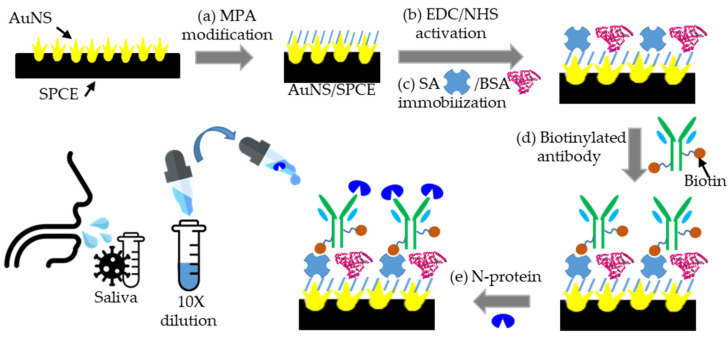
Schematic processes of the AuNS/SCPE-based immunosensor fabrication, followed by (**a**) MPA modification on the AuNS/SPCE, (**b**) EDC/NHS activation, (**c**) SA-BSA immobilization, (**d**) BioAb immobilization, and (**e**) N-protein immunoreaction.

A small amount (10 μL) of aliquot of 10 mM MPA or 10 mM MUA was dripped on the AuNS/SPCEs at 30 °C for 1 h in an incubator to form a self-assembled monolayer (SAM) as a linker for the immobilization of SA and BSA. The unbound MPA molecules were removed using double-distilled water. The carboxyl group of the MPA/AuNS/SPCEs was activated by the EDC (30 mM)/NHS (30 mM) mixture-containing 50 mM MES solution (pH 4.6) for 1 h. After rinsing the electrodes with PBS, 10 μL aliquot of SA (150 μg/mL)/BSA (150 μg/mL) mixture was placed on the activated MPA/AuNS/SPCEs or MUA/AuNS/SPCEs for 1 h. After rinsing the electrodes with PBS, the 10 μL BioAb (100 μg/mL) solution was placed on the SA-BSA-immobilized electrodes for 1 h, then dipped in the 0.0025% Tween 20-containing PBS for 10 min to remove the unbound BioAb. The biotinylation recipe of BioAb followed the Thermo company’s suggestion. Some (125 μL) aliquots of 2 mg/mL anti-SARS-CoV-2 N-protein Ab was mixed with 0.8 μL of 50 mg/mL EZ-Link Sulfo-NHS-LC-Biotin for 30 min at 25 °C, and then, 17.05 μL aliquot of 2.5 mM glycine was added to block the reaction for 1 h at 25 °C. Before use, the BioAb solution was stored at 4 °C. The immunosensors were incubated with the concentration-varied N-protein samples prepared in PBS or ten times-diluted salivary solutions for 40 min and then rinsed with PBS. EIS estimated the change in the electrochemical properties of the electrode/solution interface. The preparing procedures of the immunosensors are shown in Scheme 1.

### 2.3. Electrochemical Measurements

An equimolar Fe(CN)_6_^3−/4−^ mixture (2.5 mM) in 10 mM PBS (pH 7.4) was used as a mediator to estimate the electrochemical properties of the electrodes in each modification step and quantify the immunoreaction by an EIS workstation (MultiPalmSens4, PalmSens, Holten, The Netherlands). Some (100 μL) aliquots of the mediator-containing PBS were placed on the immunosensor surface for the EIS measurements. The EIS parameters were set in 1–100 kHz at +0.1 V versus the SPCE-based pseudo-RE, added by a 5 mV amplitude sine wave. The impedance spectra and the equivalent circuit simulation were measured using the MultiTrace-4.4 software package (PalmSens).

## 3. Results and Discussion

### 3.1. Antibody Immobilization Strategies

**Figure 1:**
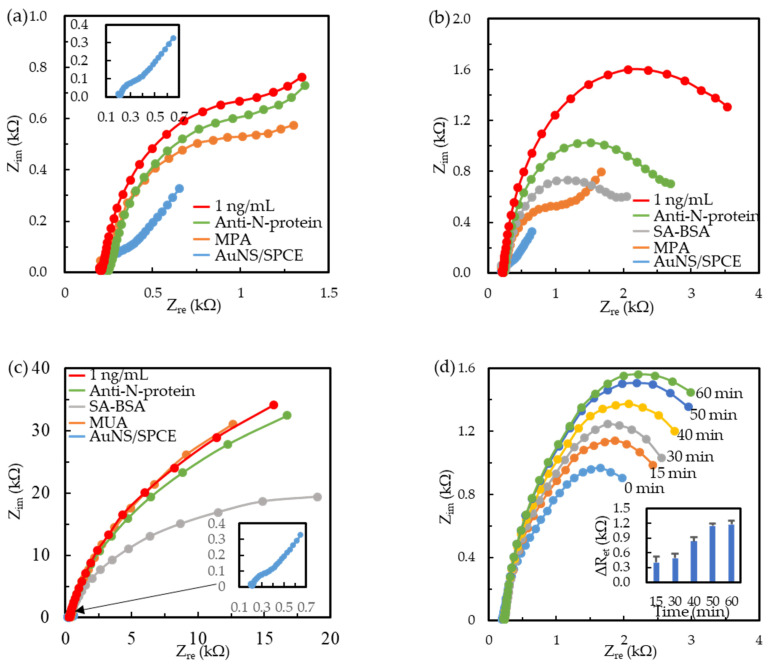
Nyquist plots of immunosensor preparation and 1 ng/mL N-protein immunoreaction of Ab/MPA/SPCE (**a**), BioAb/SA-BSA/MPA/SPCE (**b**), and BioAb/SA-BSA/MUA/SPCE (**c**) measured in a 10 mM PBS solution containing 2.5 mM equimolar Fe(CN)_6_^3−/4−^. The inset in (**a**,**c**) shows the Nyquist plot of bare AuNS/SPCEs. (**d**) Nyquist plots of time-dependent immunoreaction of 1 ng/mL N-protein on the BioAb/SA-BSA/MPA/SPCEs. Inset shows the time-dependent R_et_ increment (ΔR_et_ = R_et-N-protein_ − R_et-Ab_).

The paratope orientation of immobilized Ab affects the immunoreaction efficiency of immunosensors [43]. Figure 1 shows the Nyquist plots obtained in each step of immunosensor preparation with different immobilization strategies and their immunoreaction with 50 μL aliquot of 1 ng/mL N-protein. Figure 1a−c show the random Ab immobilization on a short-chain MPA SAM, the oriented Ab immobilization on a MPA SAM, and a long-chain MUA SAM, respectively. The inset of Figure 1a shows the impedance spectra measured at the bare AuNS/SPCEs, which exhibited a dominant linear region, implying an apparent diffusion-controlled behavior, and a small semicircle region of kinetic control attributed to the fast electron transfer rate of the Fe(CN)_6_^3−/4−^ mediator on the high conductive surface of AuNS/SPCEs [40,41,42]. Following MPA modification, the Nyquist plot showed a small linear part and a large semicircle part, implying a decreasing electron transfer rate. Moreover, the semicircle radius increased with the Ab modification and the 40-min immunoreaction of the N-protein. The phenomenon indicates that the impedance of the solution/electrode interfaces increased with the modification and the immunoreaction. The corresponding electric elements fitted by the modified Randles equivalent circuit are listed in Table 1. The modified Randles equivalent circuit, consisting of the solution resistance (R_s_), the Warburg impedance (Z_w_), the constant phase element (CPE), and the electron transfer resistance (R_et_), was mentioned in our previous articles to explain the diffusive and kinetic behavior of the solution/electrode interface [40,41,42]. The mean error of all the fitting data was less than 0.3%.

Figure 1b shows the Nyquist plots of AuNS/SPCEs, followed by MPA modification, SA-BSA immobilization, BioAb affinity attachment, and 1 ng/mL N-protein immunoreaction. The semicircle radius of the EIS plots increased with the step-by-step modification and the immunoreaction, indicating the increasing R_et_. It is worth noting that the EIS plot only presented a semicircle after the N-protein immunoreaction, implying a slow electron transfer rate to dominate the Faradaic reaction, with little diffusion-controlled behavior. In our previous studies [40,41,42], the 1R//C equivalent circuit, consisting of R_s_ in a series with one parallel circuit comprising a R_et_ and a CPE, was used to analyze the EIS spectrum of only the semicircle part. The corresponding circuit element values are statistically calculated as the mean ± standard deviation from three individual immunosensors in Table 1. The R_s_ values obtained at the Ab/MPA/SPCEs and BioAb/SA-BSA/MPA/SPCEs were almost the same when measuring the EIS in the same solution. The R_et_ values obtained in the MPA modification and the Ab immobilization of the Ab/MPA/SPCEs had no significant differences. However, the R_et_ had a significant increase, and the increment ratio (=ΔR_et_/R_et0_, ΔR_et_ = R_et-N-protein_ − R_et-Ab,_ R_et0_ = R_et-Ab_) was 27.1% after the N-protein immunoreaction. The R_et_ values obtained at the BioAb/SA-BSA/MPA/SPCEs increased with the modification steps. The ΔR_et_/R_et0_ was 77.7% after 1 ng/mL N-protein immunoreaction. Notably, the ΔR_et_/R_et0_ was 2.9 times larger than that obtained at the Ab/MPA/SPCEs, elucidating that the oriented immobilization of BioAb on the SA-BSA-modified electrodes had a higher immunoreacting efficiency than the random Ab immobilization. Many groups have demonstrated oriented Ab immobilization by using biotin–streptavidin affinity, protein A or G for the adsorption of the Ab Fc portion, or recombinant peptide tags for metal coordination with the promoted sensitivity of the immunosensors [42,43,44]. Lin et al. found that the EIS-based immunosensors with the oriented Ab immobilization on the protein A (PA, 100 μg/mL)-modified MPA/AuNS/SPCEs had a sensitivity larger than those with the random Ab immobilization [42]. Moreover, the PA (100 μg/mL)-to-BSA (100 μg/mL) ratio-modified MPA/AuNS/SPCEs could adsorb more antibodies and had a lower limit of detection (LOD) than the PA (100 μg/mL)-modified MPA/AuNS/SPCEs. Therefore, the study adopted the same concentration ratio (1:1) of SA to BSA to modify the MPA/AuNS/SPCEs. Furthermore, the MPA SAM is thin enough to produce a large CPE. The CPE value decreases significantly with the SA-BSA immobilization, Ab adsorption, and N-protein immunoreaction, attributed to layer-by-layer stacking with an increasing thickness of the modifying layer.

Figure 1c shows the EIS plots with only the semicircle part after the MUA modification. Moreover, the semicircle radius of the MUA/AuNS/SPCEs was more prominent than that of the SA-BSA- and BioAb-modified electrodes. After being analyzed by the 1R//C equivalent circuit, the R_et_ of the MUA/AuNS/SPCEs was much larger than that of the MPA/AuNS/SPCEs (shown in Table 1), resulting from the dense and long MUA SAM [41]. The high-coverage MUA COO^−^ groups presented strong electrostatic repulsion for the negatively charged Fe(CN)_6_^3−/4−^. After EDC/NHS activation and SA-BSA immobilization, the R_et_ of the SA-BSA/AuNS/SPCEs was smaller than that of the MUA/AuNS/SPCEs, attributed to the drastic decrease of the MUA COO^−^ groups. Although the ΔR_et_ (23.3 kΩ) of the BioAb/SA-BSA/MUA/SPCEs was much larger than that (1.43 kΩ) of the BioAb/SA-BSA/MPA/SPCEs after the 1 ng/mL N-protein immunoreaction, the ΔR_et_/R_et0_ (19.3%) was smaller than that (77.7%) of the BioAb/SA-BSA/MPA/SPCEs. The results elucidated that the MPA linker and SA-BSA layer combination can produce a more sensitive immunosensor, and the ΔR_et_/R_et0_ is more significant than the ΔR_et_ for EIS signal compared between fabrication-varied immunosensors. Figure 1d shows the time-dependent immunoreaction curve. The ΔR_et_ values measured at 50 min reached a saturated reaction. We wanted to compromise the time and response magnitude of the immunoreaction to select a 40-min immunoreaction to investigate other sensing properties.

### 3.2. Calibration Curves

**Figure 2:**
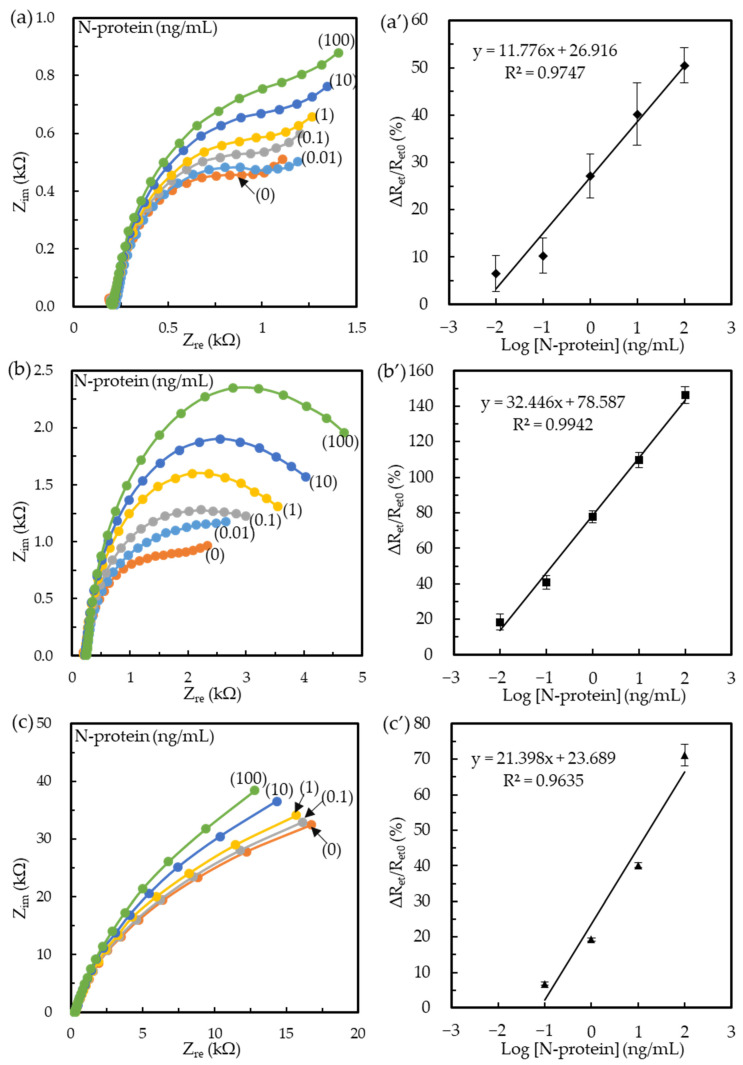
Nyquist plots of the (**a**) Ab/MPA/SPCE, (**b**) BioAb/SA-BSA/MPA/SPCE, and (**c**) BioAb/SA-BSA/MUA/SPCE-based immunosensors after sequentially immunoreacting with the N-protein samples of 0–100 ng/mL concentrations. Curves (i–vi) are 0 (i), 0.01 (ii), 0.1 (iii), 1 (iv), 10 (v), and 100 ng/mL (vi) N-protein, respectively. (**a’**–**c’**) The calibration curves corresponding to (**a**–**c**). (*n* = 3).

Figure 2 shows the EIS plots obtained at the three kinds of immunosensors after N-protein immunoreaction and their corresponding calibration curves. The results of Figure 2a–c showed that the semicircle radius increased with the increasing N-protein concentration. Each calibration curve of ΔR_et_/R_et0_ versus N-protein concentration was statistically calculated from three individual immunosensors with error bars to mark one standard deviation. Figure 2a’ shows the linear regression equation of the Ab/MPA/SPCEs as ΔR_et_/R_et0_ (%) = 11.776 log[N-protein] (ng/mL) + 26.916 in the dynamic range of 0.01−100 ng/mL. The calculated LOD was 46 pg/mL (S/N > 3). Furthermore, the original R_et_ standard deviation and mean value were used to calculate relative standard deviations (RSD). The RSD of the Ab/MPA/SPCEs obtained in 0.01−100 ng/mL was between 3.7% and 6.5%. Figure 2b’ shows the linear equation of the BioAb/SA-BSA/MPA/SPCEs as ΔR_et_/R_et0_ (%) = 32.446 log[N-protein] (ng/mL) + 78.587. The linear range was 0.01−100 ng/mL, with a correlation coefficient (R) of 0.997, indicating excellent linearity. The calculated LOD was 6 pg/mL (S/N > 3). The R_et_ RSD in the detection range of 0.01−100 ng/mL was 3.3–4.9%. The small RSD implies the high reproducibility of the SA-BSA-based immunosensors, attributed to the good control of the paratope orientation. Figure 2c’ shows the regression equation of the BioAb/SA-BSA/MUA/SPCEs as ΔR_et_/R_et0_ (%) = 21.398 log[N-protein] (ng/mL) + 23.689. The linear range was only in the range of 0.1−100 ng/mL. The calculated LOD was 174 pg/mL (S/N > 3), higher than that of the MPA-based immunosensors. This phenomenon was attributed to the high insulation and thickness of the MUA SAM to reduce the electric response in the Ab–antigen interface [41]. Moreover, the RSD of the calibration curve ranged from 0.4% to 3.1%, implying a high reproducibility of the MUA-based immunosensors.

### 3.3. Other Sensing Properties

**Figure 3:**
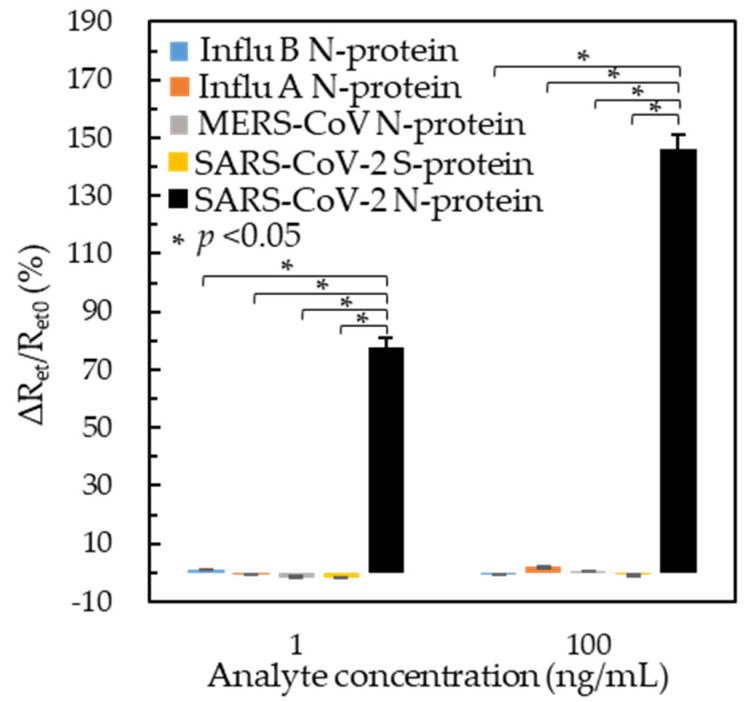
Specificity test of the BioAb/SA-BSA/MPA/SPCEs of different analytes in PBS. The data of the SARS-CoV-2 N-protein are retrieved from Figure 2b’ for comparison of the cross-reactivity measured by three individual sensors. The asterisk indicates a significant difference (*p* < 0.05).

The BioAb/SA-BSA/MPA/SPCE-based immunosensors were tested against different viral antigens, including the MERS-CoV N-protein, influenza A virus N-protein, influenza B virus N-protein, and SARS-CoV-2 S-protein, to investigate the immunosensor specificity. Figure 3 shows the ΔR_et_/R_et0_ response of each analyte in low (1 ng/mL) and high (100 ng/mL) concentrations. Compared to the ΔR_et_/R_et0_ values of 1 ng/mL (77.7%) and 100 ng/mL (146.2%) SARS-CoV-2 N-protein, the ΔR_et_/R_et0_ values of the other analytes ranged from 0.51% to 1.21% and from 0.58% to 1.84%, respectively; those are much smaller than the ΔR_et_/R_et0_ of SARS-CoV-2 N-protein. The signal-to-cross reactivity ratio of 1 ng/mL and 100 ng/mL SARS-CoV-2 N protein was larger than 64.2 and 79.5, respectively. The results indicated that the developed immunosensors have excellent specificity for the SARS-CoV-2 N-protein and little cross-reactivity for the MERS-CoV N-protein, influenza A virus N-protein, influenza B virus N-protein, and SARS-CoV-2 S-protein.

### 3.4. Saliva-Based Tests

**Figure 4:**
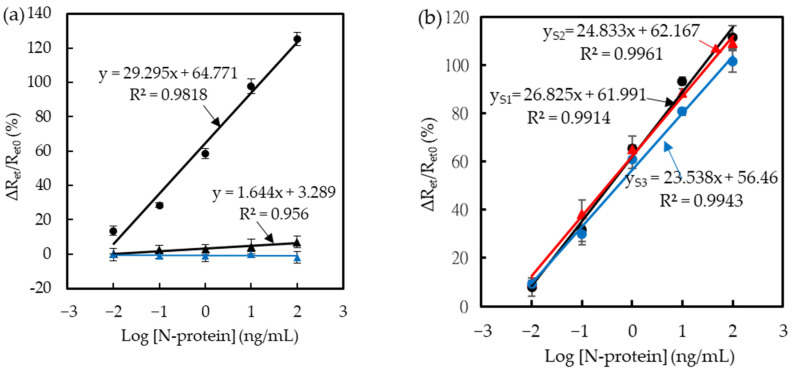
Effect of saliva on the sensing properties of BioAb/SA-BSA/MPA/SPCE-based immunosensors. Each curve was obtained from three individual immunosensors. (**a**) The black circle, the black triangle, and the blue triangle indicate the EIS measurements in SARS-CoV-2-spiked saliva, diluted saliva, and blank PBS, respectively. (**b**) The ΔR_et_/R_et0_ response measured from three individual sensors in the N-protein-spiked volunteer salivary samples. Black, red, and blue lines indicate the regression curves of the S1, S2, and S3 sensors, respectively.

To validate the detecting ability of the immunosensors for COVID-19 diagnostics in a more realistic scenario, the measurements were taken from commercial saliva to mimic the actual samples. Saliva is viscous and tends to congeal after collection, making it difficult to be accurately pipette for liquid-based manipulation. Therefore, PBS was used to mix with saliva to lower the viscosity. Previous studies showed that the SARS-CoV-2 N-protein could be directly detected in PBS-diluted saliva for COVID-19 rapid tests [11,39]. Following the PBS-based immunoassay, the ten times-diluted salivary solutions were spiked with SARS-CoV-2 N-protein. Figure 4 shows the ΔR_et_/R_et0_ results in the diluted saliva with and without N-protein. The linear regression equation of N-protein detection was ΔR_et_/R_et0_ (%) = 29.295 log[N-protein] (ng/mL) + 64.771 in the dynamic range of 0.01−100 ng/mL. The correlation coefficient was 0.991, implying good linearity. The calculated LOD was 6 pg/mL (S/N > 3), the same as the LOD obtained in PBS. The slope (29.30% mL/ng) of the calibration curve was slightly smaller than that (32.45%∙mL/ng), attributed to the effect of the saliva viscosity on the immunoreaction efficiency [22]. Furthermore, the immunosensors were tested with the same immunoreacting procedures in SARS-CoV-2 N-protein-free saliva and PBS, as shown in Figure 4a. The repetition test in blank solutions can realize the short-term stability of immunosensors and the effect of the interferents existing in saliva on the sensors. The ΔR_et_/R_et0_ values increased slightly with the repetition number measured in the N-protein-free saliva samples, and the slope of the linear regression curve was 1.644 (%∙mL/ng), which was only 0.056 times smaller than that obtained in the N-protein-containing saliva samples. The ΔR_et_/R_et0_ difference between the 0.01−100 ng/mL N-protein-containing saliva and the N-protein-free saliva was significant (*p* < 0.05) (*t*-test analysis). This result implies that the influence of a saliva substrate on the sensing result can be ignored. Compared to the N-protein-free saliva, the ΔR_et_/R_et0_ measured in blank PBS ranged from −0.1% to −1.7%, indicating that the sensor stability was good after repeatedly dripping blank PBS and mediator-containing PBS for the background test and the EIS measurements.

Furthermore, four healthy volunteers donated their saliva for real sample testing instead of patient saliva. First, the concentration-varied N-protein was spiked in the 10 times-diluted human salivary solutions, and then, the salivary solutions were filtered through 0.22-μm pore size syringe filters (PES membrane, Millex-GP, Merck Millipore Ltd., Dublin, Ireland). Some (50 μL) aliquots of the salivary filtrate were dripped on the immunosensors for 40 min, and then, PBS was used to rinse the immunosensors. Subsequently, the 2.5 mM Fe(CN)_6_^3−/4^-containing PBS was dripped on the immunosensors for the EIS measurements. Figure 4b shows the ΔR_et_/R_et0_ signal obtained from three individual immunosensors, called S1, S2, and S3. The results showed that the three immunosensors had good linearity in 0.01−100 ng/mL. The LOD values obtained at the S1, S2, and S3 sensors were 6, 5, and 7 pg/mL. The results suggest that the constructed immunosensors are still feasible for real-saliva detection. Furthermore, the sensitivity of the S1–S3 sensor regression curves was smaller than that obtained in the diluted artificial saliva, attributed to the composition and viscosity of different volunteer saliva.

The methodology, preparing strategies, and sensing properties of the immunosensors are compared with other electrochemical affinity-based sensors in Table 2. Generally, the nanomaterial-modified immunosensors have promising potential to obtain a lower LOD [25,26,45]. Our BioAb/SA-BSA/MPA/SPCE-based immunosensor presented a low LOD, a wide linear range, and a simple label-free sensing strategy. Some studies and this work can reach pg/mL-scaled LOD [24,25,26,32,45]. Shan’s clinical study found that, when the PCR Ct value was higher than 30, the corresponding N-protein concentration in saliva was lower than pg/mL [39]. Therefore, the future mission is to promote the LOD of immunosensors for more sensitive saliva-based COVID-19 antigen testing.

## 4. Conclusions

In this work, we constructed BioAb/SA-BSA/MPA/AuNS/SPCE-based immunosensors for the label-free detection of the SARS-CoV-2 N-protein in saliva samples. The MPA SAM for the SA-BSA immobilization allows EIS-based immunosensors to have a more sensitive response to the antigen-Ab immunoreaction than the MUA SAM. The oriented adsorption of BioAb on the SA-BSA layer can facilitate the sensitivity and reproducibility of the fabricated immunosensors. The immunosensors presented good linearity from 0.01 ng/mL to 100 ng/mL and a low LOD of 6 pg/mL in the diluted saliva. Moreover, the high signal-to-cross reactivity ratio of the immunosensors implies excellent specificity of the SARS-CoV-2 N-protein, indicating that the surface modification technique can effectively reduce the nonspecific adsorption of other viral antigens. The saliva-based immunosensor exhibits promising potential to develop a rapid and easy operational device for COVID-19 diagnostics.

## Figures and Tables

**Table 1:** The electric element values fitted from the experimental spectra of Figure 1a–c. Each data is calculated from three individual electrodes.

Modification Steps	Ab/MPA/SPCEs			BioAb/SA-BSA/MPA/SPCEs			BioAb/SA-BSA/MUA/SPCEs		
	R_s_ (kΩ)	R_et_ (kΩ)	CPE (μF)	R_s_ (kΩ)	R_et_ (kΩ)	CPE (μF)	R_s_ (kΩ)	R_et_ (kΩ)	CPE (μF)
SAM	0.23 ± 0.00	1.15 ± 0.03	10.49 ± 0.03	0.23 ± 0.00	1.23 ± 0.03	10.07 ± 0.03	0.35 ± 0.01	188.3 ± 16.0	2.44 ± 0.01
SA-BSA	.	.	.	0.23 ± 0.00	1.45 ± 0.03	9.17 ± 0.05	0.29 ± 0.00	51.9 ± 0.9	2.32 ± 0.02
Ab	0.22 ± 0.00	1.07 ± 0.04	10.19 ± 0.10	0.22 ± 0.00	1.84 ± 0.04	8.65 ± 0.02	0.28 ± 0.00	120.9 ± 3.0	2.23 ± 0.01
N-protein	0.22 ± 0.00	1.36 ± 0.05	10.95 ± 0.06	0.24 ± 0.00	3.27 ± 0.06	6.64 ± 0.00	0.29 ± 0.00	144.2 ± 0.5	2.24 ± 0.00

**Table 2:** Sensing properties and detecting strategies of SARS-CoV-2 N-protein electrochemical immunosensors between this study and previous research.

Methodology	Electrode Modification	Detecting Procedures/Testing Time	Linear Range	LOD (pg/mL)/Solution	Ref.
EGFET ^1^	Thiolated CAb/AuE ^2^	Label-free (<30 min)	0.4−400 ng/mL	340/PBS 140/2× saliva	[22]
Amperometry	Thiolated CAb/AuE	HRP-DAb@MB ^3^, N-protein, and CAb/AuE in sandwich type (<60 min)	1−10 ng/mL	50/serum 10/5× serum	[23]
DPV	Carbon black/SPCE	DAb-RAb-HPR ^4^, N-protein, and CAb@MAb/MB ^5^ in sandwich type (30 min)	100−1000 ng/mL	8000/PBS & untreated saliva	[17]
DPV	MIP ^6^/AuE	Label-free (15 min)	0.1−5.9 pg/mL	0.8/PBS 1.4/20× NPGL ^7^	[24]
DPV	Aptamer/AuE	HRP-Hermin@Au-Pt/MIL-53 ^8^, N-protein, and aptamer/AuE in sandwich type (60 min)	0.025−50 ng/mL	8.3/PBS 93.8/diluted serum	[32]
SWV	CAb/MUA/AuNP/SPCE	Label-free (15 min)	0.001–100 ng/mL	0.4/PBS	[25]
SWV	N-protein/carbon nanofiber/SPCE	N-protein Ab, N-protein, and the immunosensor in competition type (20 min)	1–1000 ng/mL	0.8/PBS	[45]
EIS	Aptamer/Nanodiamond/AuE	Label-free (5 min)	0.05 pg/mL–5 ng/mL	0.02/PBS 0.02/10× serum	[26]
EIS	BioAb/SA-BSA/MPA/SPCEs	Label-free (40 min)	0.01−100 ng/mL 0.01−100 ng/mL	6/PBS 6/10× saliva	This work

^1^ EGFET: extended gate field effect transistor; ^2^ CAb: capture Ab; ^3^ HRP-DAb@MB: horseradish peroxidase-conjugated detection Ab-modified magnetic bead; ^4^ DAb-RAb-HPR: DAb immunoreacted with HRP-conjugated rabbit Ab; ^5^ CAb@MAb/MB: CAb immunoreacted with mouse Ab-modified MB; ^6^ MIP: molecularly imprinted polymer; ^7^ NPGL: nasopharyngeal liquid; ^8^ MIL-53: one of the metal–organic frameworks.

## Data Availability

Not applicable.
